# Hemagglutinin-specific neutralization of subacute sclerosing panencephalitis viruses

**DOI:** 10.1371/journal.pone.0192245

**Published:** 2018-02-21

**Authors:** Miguel Ángel Muñoz-Alía, Claude P. Muller, Stephen J. Russell

**Affiliations:** 1 Department of Molecular Medicine, Mayo Clinic, Rochester, Minnesota, United States of America; 2 Department of Infection and Immunity, Luxembourg Institute of Health, Esch-Sur-Alzette (Grand Duchy of Luxembourg), Luxembourg; 3 Laboratoire National de Santé, Dudelange, Luxembourg; 4 Division of Hematology, Mayo Clinic, Rochester, Minnesota, United States of America; Deutsches Primatenzentrum GmbH - Leibniz-Institut fur Primatenforschung, GERMANY

## Abstract

Subacute sclerosing panencephalitis (SSPE) is a progressive, lethal complication of measles caused by particular mutants of measles virus (MeV) that persist in the brain despite high levels of neutralizing antibodies. We addressed the hypothesis that antigenic drift is involved in the pathogenetic mechanism of SSPE by analyzing antigenic alterations in the MeV envelope hemagglutinin protein (MeV-H) found in patients with SSPE in relation to major circulating MeV genotypes. To this aim, we obtained cDNA for the MeV-H gene from tissue taken at brain autopsy from 3 deceased persons with SSPE who had short (3–4 months, SMa79), average (3.5 years, SMa84), and long (18 years, SMa94) disease courses. Recombinant MeVs with a substituted MeV-H gene were generated by a reverse genetic system. Virus neutralization assays with a panel of anti-MeV-H murine monoclonal antibodies (mAbs) or vaccine-immunized mouse anti-MeV-H polyclonal sera were performed to determine the antigenic relatedness. Functional and receptor-binding analysis of the SSPE MeV-H showed activity in a SLAM/nectin-4–dependent manner. Similar to our panel of wild-type viruses, our SSPE viruses showed an altered antigenic profile. Genotypes A, G3, and F (SSPE case SMa79) were the exception, with an intact antigenic structure. Genotypes D7 and F (SSPE SMa79) showed enhanced neutralization by mAbs targeting antigenic site IIa. Genotypes H1 and the recently reported D4.2 were the most antigenically altered genotypes. Epitope mapping of neutralizing mAbs BH015 and BH130 reveal a new antigenic site on MeV-H, which we designated Φ for its intermediate position between previously defined antigenic sites Ia and Ib. We conclude that SSPE-causing viruses show similar antigenic properties to currently circulating MeV genotypes. The absence of a direct correlation between antigenic changes and predisposition of a certain genotype to cause SSPE does not lend support to the proposed antigenic drift as a pathogenetic mechanism in SSPE.

## Introduction

Measles virus (MeV) is a single-stranded, negative-sense RNA virus, a member of the Paramyxoviridae family, genus *Morbillivirus*, and is the causative agent of measles [1]. The envelope hemagglutinin glycoprotein on MeV interacts with cellular receptors, which determines the virus’ pathogenesis. The virus initiates infection in the respiratory tract by infecting resident immune cells expressing the receptor signaling lymphocytic activation molecule (SLAM)/CD150 [2,3]. Next, MeV is transferred to local lymph nodes and spreads systemically to other organs and tissues by cell fusion, forming syncytia, known as Warthin-Finkeldey cells. Airborne transmission results from coughing and sneezing induced by damage to MeV-infected, nectin-4/PVRL4–positive cells in the epithelium [4,5].

The intrinsic lymphotropic nature of MeV produces a transient and severe immunosuppression. The most common measles complications are MeV interstitial pneumonitis and giant cell pneumonia, otitis media, and diarrhea [6]. Although MeV is not a highly neurotropic virus, 1 in 1,000 patients will have lethal central nervous system complications in the form of acute measles postinfection disseminated encephalomyelitis, measles inclusion-body encephalitis (in immunocompromised persons), and subacute sclerosing panencephalitis (SSPE) [7,8]. SSPE is a fatal complication of MeV infection that typically arises 1 to 15 years after acute measles. The incidence of SSPE has been recently reported as high as 1 in 1,367 for children younger than 5 years in the United States [9].

SSPE is caused by the same common acute MeV strain that, from a single point of entry, invades and progressively disseminates throughout the brain [10]. The virus that is present at the onset of SSPE symptoms, however, differs substantially from the original MeV strain and is referred to as *mutant* or *SSPE virus*. An abnormally high concentration of MeV-specific neutralizing antibodies in the serum and cerebrospinal fluid is a hallmark of SSPE diagnosis [11]. In spite of this, however, the immune response is ineffective in clearing the virus from the brain [12].

It has long been suggested that antigenic drift has a role in SSPE pathogenesis. The fact that wild-type genotypes show a distinct signature of antigenic determinants in comparison to genotype A prompted us to review the long-held hypothesis of antigenic drift in viral persistence. Because the MeV envelope hemagglutinin protein (MeV-H) is the major target of virus-neutralizing antibodies that arise during infection by MeV and because it is subject to increased immunologic pressure [13–17], we generated viruses that contained the MeV-H genes found in 3 SSPE autopsy cases. We aimed to investigate the hypothesis that antigenic drift is involved in the pathogenesis of SSPE by analyzing antigenic alterations in MeV-H found in decedents with SSPE in relation to major circulating MeV genotypes.

## Results

### Mutational burden in MeV-H

Because protection from MeV infection correlates best with anti-MeV-H antibodies, we focused our analysis on this glycoprotein in brain autopsy material from 3 decedents with SSPE from Madrid, Spain [7,18]. The SSPE disease course in these 3 patients was either short (3 months; patient designated SMa79), average (3.5 years; SMa84), or long (18 years; SMa94) (Table 1). The MeV-H amino acid sequences for SMa84 (Z80810.1) and SMa94 (Z80830.1) have already been reported [19]. The SMa79 MeV-H clone had a series of amino acid changes that differed from the consensus sequence (Z80828.1) at several positions: D14G, I65T, L233I, E492G, and D574N (Table 2).

**Table 1:** Characteristics of subacute sclerosing panencephalitis cases.

	Decedent		
Characteristic	SMa94	SMa79	SMa84
Geographical origin	Madrid (Spain)	Madrid (Spain)	Madrid (Spain)
Sex	Female	Male	Male
Year of birth	1967	1965	1968
Acute measles	1968	1967	1970
Onset of neurologic symptoms	1977	June 1979	September 1981
Date of death	May 1994	September 1979	March 1984
Length of clinical symptoms	17 years	3 months	3.5 years
Measles virus genotype	F	F	C1

**Table 2:** Amino acid sequence comparison of MeV-H from the 3 SSPE cases and the Moraten vaccine strain^a^.

	MeV-H			
Amino Acid Position	Moraten Vaccine (AF266287)	SMa79 (Z80828)	SMa84 (Z80810)	SMa94 (Z80830)
8	I	T	●	●
11	F	L	●	●
14	D	*G*	●	●
29	H	●	Y	●
48	S	●	G	●
62	R	Q	●	Q
65	I	*T*	●	●
100	G	●	S	●
159	A	●	V	●
177	T	●	A	●
180	F	●	S	●
181	L	●	P	●
200	N	●	D	●
210	L	●	●	V
233	L	*I*	●	●
243	R	G	G	G
276	L	F	F	F
283	D	●	●	G
302	G	●	●	W
382	F	●	●	S
394	C	●	Y	●
396	N	D	●	●
412	V	●	F	●
423	L	●	P	F
481	Y	N	N	N
492	E	*G*	●	●
547	R	●	●	S
574	D	*N*	●	●
575	Q	●	K	K
614	T	●	●	I
615	N	S	●	●
618	STOP	W	●	Q
619	…	G	●	G
620	…	C	●	C
621	…	Q	●	Q

Abbreviations: MeV-H, measles virus envelope hemagglutinin protein; SSPE, subacute sclerosing panencephalitis.

^a^ Only amino acids that differ from those in the Moraten vaccine strain are shown. Identical residues are indicated by dots. Amino acids that do not represent the consensus sequence are shown in italics.

**Fig 1:**
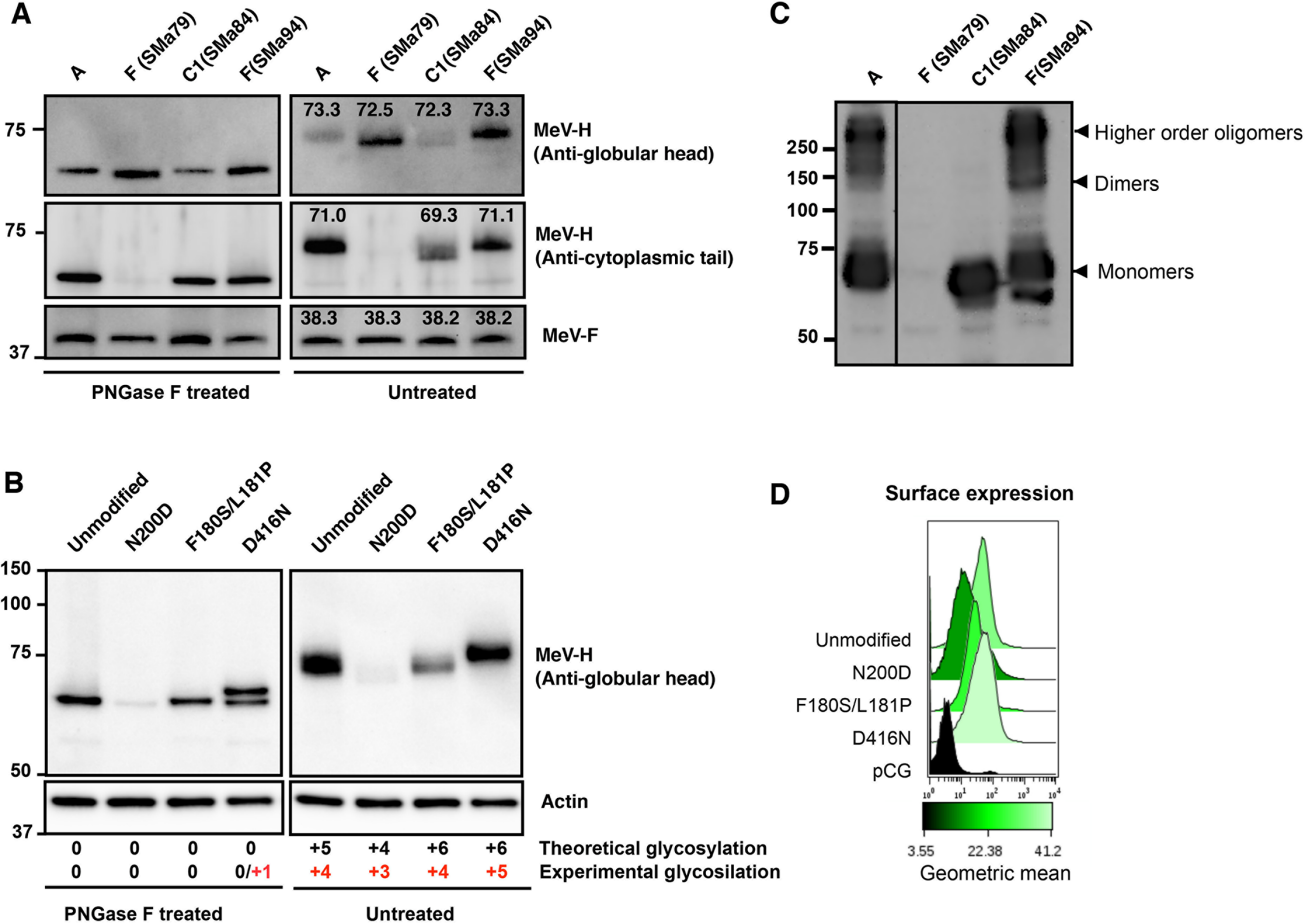
Glycosylation and Oligomerization state of SSPE MeV-H. A, Electrophoretic mobility of PNGase F-treated or untreated SSPE MeV-H proteins. Cytoplasmic extract of Vero cells transfected with plasmid DNA encoding MeV-F protein from genotype A and different MeV-H forms (genotype A; genotype F, patients SMa79 and SMa94; and genotype C1, patient SMa84) were treated with or without PNGase F before they were immunoblotted with the indicated antibodies. MeV-F served as a double control for transfection and gel electrophoresis. In the presence of PNGase F, the electrophoretic migration pattern is not affected by glycosylation status. The apparent molecular mass is indicated above the corresponding band for the untreated MeV-H. MeV-H genotype C1 lacks a potential N-linked glycosylation site (PNGS) at N200 and shows a downward band shift compared with MeV-H genotype A and F (SMa94) proteins. MeV-H F (SMa79) cannot be detected by anti-MeV-H cytoplasmic tail antibodies, but the anti-MeV-H globular head BH195 antibody revealed a similar downward band shift regardless of the presence of N-linked glycans. B, Glycosylation of MeV-H mutants. MeV-H encodes 5 PNGS at N168, N187, N200, N215, and N238, but only the first 4 have been shown to be glycosylated (theoretical vs experimental glycosylation). An N200D mutation would destroy an active PNGS, whereas F180S/L181P and D416N would create additional PNGSs. A downward shift is observed for N200D, whereas an upward shift is seen for the D416N mutant. No changes in electrophoretic mobility were observed for the F180S/L181P mutant. After removal of N-linked glycans, all proteins migrate equally. D416N shows an additional band likely corresponding to a +1 PNGS. C, Oligomerization state of SSPE MeV-H proteins. Cytoplasmic extracts were resolved under nonreducing conditions and immunoblotted with anti-MeV-H_cyt_. The different oligomeric states are indicated. D, Surface expression of the mutant MeV-H proteins. Cell surface expression was monitored by flow cytometry after transfection with pCG-MeV-H (genotype A) expression plasmid and the mutants used in panel B. Empty pCG plasmid was used as negative control. MeV-H protein surface expression was detected with anti-MeV-H BH026 antibody followed by goat anti-mouse IgG (H+L) cross-adsorbed secondary antibody, Alexa Fluor 594.

The predicted MeV-H amino acid sequence of the 2 SSPE cases that belong to genotype F (SMa79 and SMa94) is extended by 4 amino acids. In addition, MeV-H from SMa79 has 3 mutations in the N-terminal cytoplasmic tail (I8T, F11L, and D14G). Indeed, of the 3 SSPE cases, only MeV-H of SMa79 could not be detected on Western blotting with antibodies to the MeV-H cytoplasmic tail (Fig 1A).

The amino acid sequence of MeV-H from SMa84, which is of genotype C1, predicts the lack of an N-linked carbohydrate at N200, because of an N200D point mutation (Table 2). Of the potential N-linked glycosylation sites (PNGSs) of MeV-H, N200 is known to be glycosylated [20,21]. In addition, an F180S point mutation in this MeV-H SMa84 creates a PNGS at N178 (178NQF→NQS), although its potential usage was not predicted by the NetNGlyc 1.0 server [22]. Thus, we investigated the glycosylation status of MeV-H SMa84 by peptide-N-glycosidase F (PNGase F) treatment, which releases asparagine-linked oligosaccharides. In the presence of PNGase F, all MeV-H forms were completely deglycosylated and showed an identical migration pattern, with the exception of MeV-H SMa79 that migrated at a slightly lower molecular weight (Fig 1A). When the proteins were left untreated, the electrophoretic mobility of the MeV-H SMa79 and SMa84 was decreased in relation to that of MeV-H A and SMa94. These results indicate that an N-glycan moiety is absent in the MeV-H of genotype C1 (SMa84), whereas the precise biochemical aberration causing a lower molecular weight for SMa79 is currently unknown.

To further confirm the lack of usage of the PNGS 178NQS, we introduced the F180S mutation in the MeV-H from genotype A. We also included the L181F mutation, which is present in SMa84, because the N-glycosylation efficiency is dependent on the surrounding sequence [23]. Previously, Santibañez and coworkers [24] confirmed the glycosylation of a PNGS 416DLS>NLS in genotype D7. We therefore introduced a D416N mutation as a positive control for our experimental setup. Total and surface expression levels were decreased for the N200D mutant but were comparable in the other mutants (Fig 1B and 1D). Curiously, a distinct double band in the MeV-H D416N was observed after PNGase F treatment (apparent molecular masses of 59.3 and 61.3 kDa) (Fig 1B). This higher-molecular-weight band most likely corresponds to a partially deglycosylated form. As expected, the relative mobility of the D416N mutant was decreased compared with the unmodified/unmutated MeV-H, but it was increased for the N200D mutant (Fig 1B). No differences in electrophoretic mobility were observed for the F180S/L181P mutant. Overall, these results confirm that an N-glycan moiety is absent in the MeV-H of genotype C1. Interestingly, the gel shift was greater in the MeV-H D416N mutant than in genotype C1 (SMa84) (mean [SD]: 3.65[0.21] kDa vs 1.60 [0.14] kDa), despite both types being distinguishable from the standard MeV-H genotype A by 1 glycan moiety (Fig 1).

Because glycan processing has been correlated with MeV-H dimer formation [21], we determined the oligomeric state of MeV-H genotype C1 by analyzing the same samples as in Fig 1A under nonreducing conditions. Whereas MeV-H of genotype A and F (SMa94) showed bands corresponding to monomers, dimers, and higher-order oligomers, MeV-H SMa84 was almost exclusively present as monomers (Fig 1C).

### Functional analysis of MeV-H from SSPE cases

**Fig 2:**
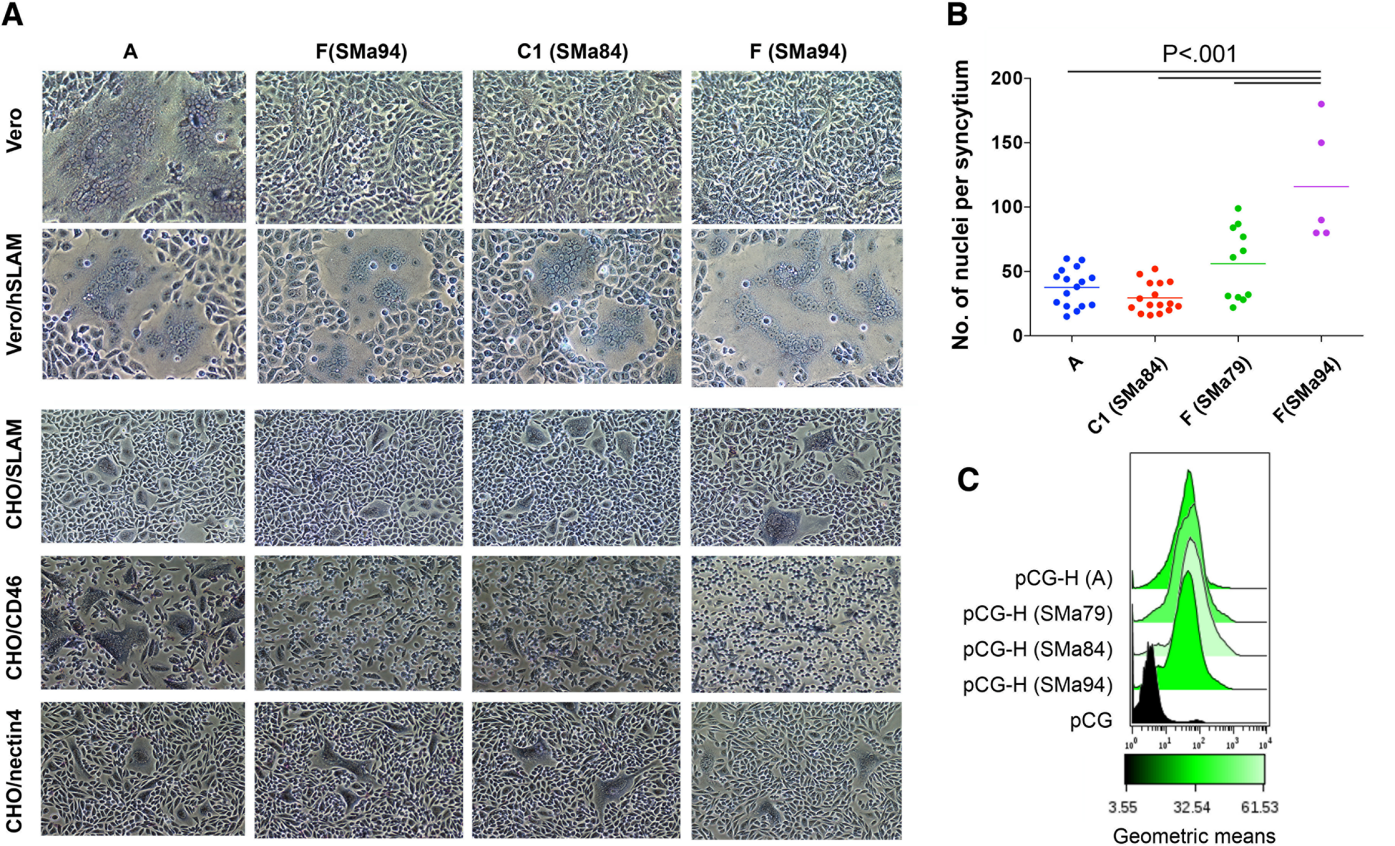
Fusion activity of SSPE MeV-H. A, Vero, Vero/hSLAM, CHO/SLAM, CHO/CD46, and CHO/nectin-4 cells were cotransfected with DNA expression plasmids for the vaccine MeV-F and the MeV-H indicated at the top. At 24 hours, cells were stained and micrographs were taken at ×4 magnification. B, Fusion activity observed in Vero/hSLAM cells was quantitated by counting the number of nuclei in randomly chosen syncytia. Statistical probabilities, calculated by ANOVA, are indicated. C, Surface expression of the SSPE MeV-H proteins. Levels of cell surface expression were determined as in Fig 1D.

Because SSPE viruses are characterized by mutations that apparently lead to defective budding and absence of syncytia [8], we evaluated the functionality of the SSPE MeV-H forms by fusion assay. Cotransfection of expression plasmids for MeV-H and the MeV envelope fusion glycoprotein (MeV-F) genes results in cell fusion visualized as syncytia. When we transfected plasmids encoding different SSPE MeV-Hs in combination with MeV-F of genotype A, syncytia were detected in Vero cells expressing human SLAM (Vero/hSLAM) but not in the wild-type Vero cell line. Only MeV-H genotype A induced syncytia in Vero cells (Fig 2A). In this fusion assay, the fusion efficiency of MeV-H SMa94 was the greatest; quantifying the fusion activity was difficult because of the extent of syncytium formation (Fig 2B). Increased surface expression in MeV-H SMa94 was excluded as a potential reason for the enhanced fusogenicity because all MeV-H proteins showed comparable efficiency of surface expression (Fig 2C).

To investigate whether cell fusion occurred in a receptor-specific manner, we next examined the ability of SSPE MeV-H species to induce fusion in Chinese hamster ovary (CHO) cells expressing the MeV receptors. Syncytia were observed in CHO cells expressing CD46 only when MeV-H genotype A was used. Rather than showing defects in syncytium-inducing capacity, the extent of syncytium-forming activity for MeV-H SMa94 was greater in a SLAM-dependent manner. No noticeable differences in fusogenicity were observed in CHO cells expressing nectin-4 (Fig 2A).

**Fig 3:**
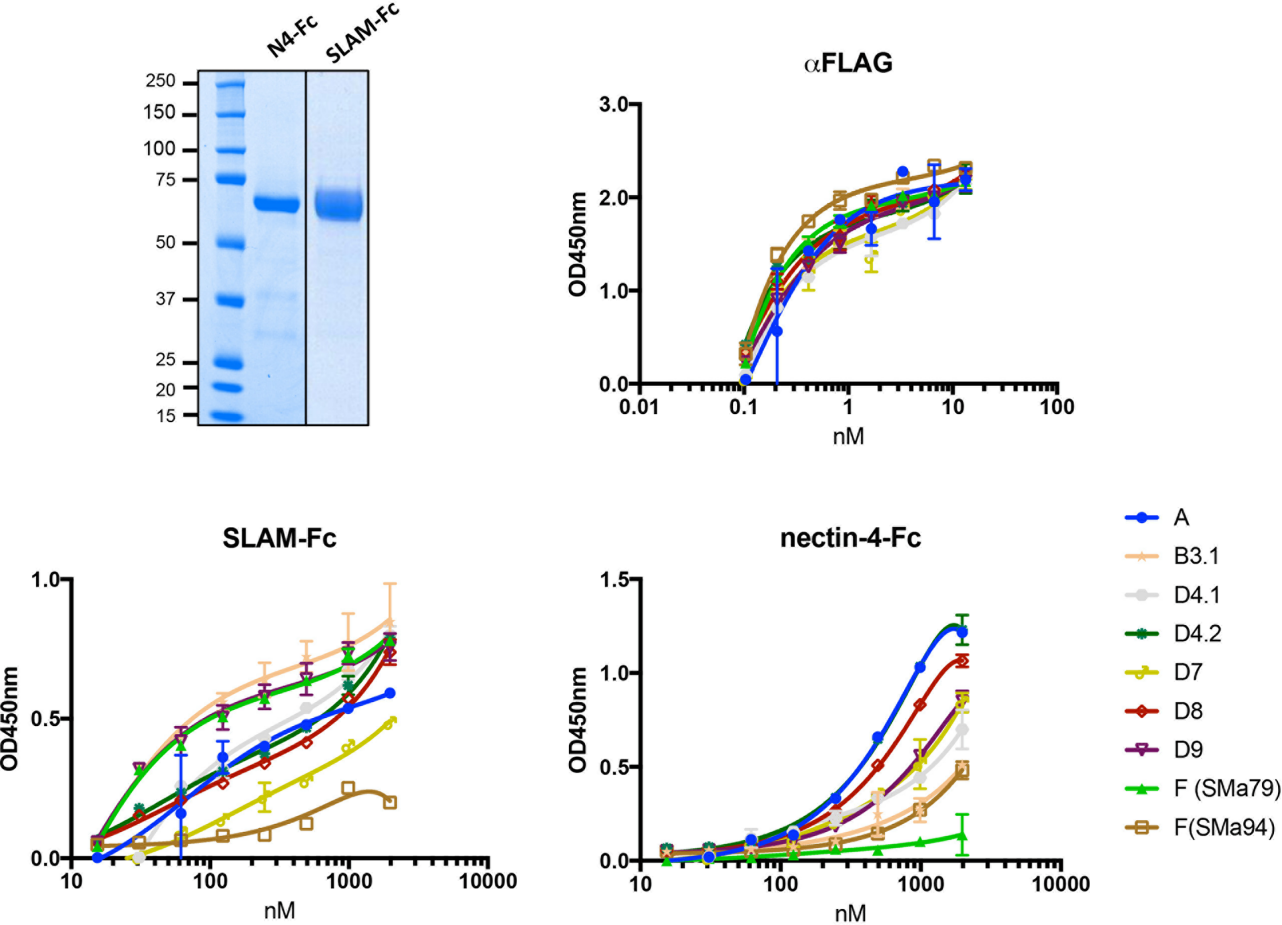
Receptor interaction of MeV-Hs. Upper left panel: Protein-G–purified soluble MeV receptor-Fcs were separated by SDS-PAGE under reducing conditions and visualized by Coomassie brilliant blue staining. Upper right and lower panels: Binding of soluble MeV-Fc receptors to plastic-bound MeV-H. Binding was monitored by optical density (OD) (see Methods).

The above results show that SSPE MeV-H can mediate fusion triggered by interacting with cellular receptors. Because of the above differences in the cell-to-cell fusion activity, we assessed the interaction of the MeV-Hs with their receptors SLAM and nectin-4 (Fig 3). For this experiment, we included other wild-type MeV-Hs from major circulating MeV genotypes. Unfortunately, we were unable to express and purify useful amounts of MeV-H SMa84. Soluble protein fragments of SLAM and nectin-4 were produced having their respective whole extracellular domains. The IgG_1_-Fc region fused to the receptors was used for purification via protein G, which facilitated detection of the protein bound to the plastic-coated MeV-H. Soluble MeV-Hs were produced carrying a Strep-tag II epitope in the N-terminal domain to be captured in a MeV-H-nativelike conformation on the surface of plastic wells. A FLAG-tag epitope in the C-terminal domain was also attached to control the amount of protein used for coating. Binding of anti-FLAG antibody to the plastic-coated MeV-H increased with antibody concentration and was similar for all MeV-H forms tested, which indicated similar amounts of coated protein per well (Fig 3).

When receptor-Fc fusion proteins were used, the shapes of the saturation curves differed. MeV-H from genotypes B3.1, D9, and F (SMa79) was saturated at an 8-fold lower concentration of SLAM receptor than genotypes D4.2 and D8 (Fig 3). Only at concentrations higher than 2,000 nM were binding values indistinguishable among the MeV-H forms tested. Surprisingly, MeV-H genotype F (SMa94) showed only residual binding to SLAM, but its binding to nectin-4 was similar to that of genotype B3.1. According to the nectin-4 binding curve shape, a low-binding-affinity group comprising genotypes F (SMa94) and B3.1 was identified. However, the other MeV-H from genotype F, SMa79, showed only residual binding to nectin-4. These results showed that even though specific MeV-F and MeV-H interactions are required for efficient fusion, MeV-H from SSPE strains can cooperate efficiently with MeV-F genotype A to trigger cell fusion. However, discrepancies exist between functional and biochemical analyses, as previously reported [25].

### Syncytium-forming activity of recombinant MeV encoding SSPE MeV-H

**Fig 4:**
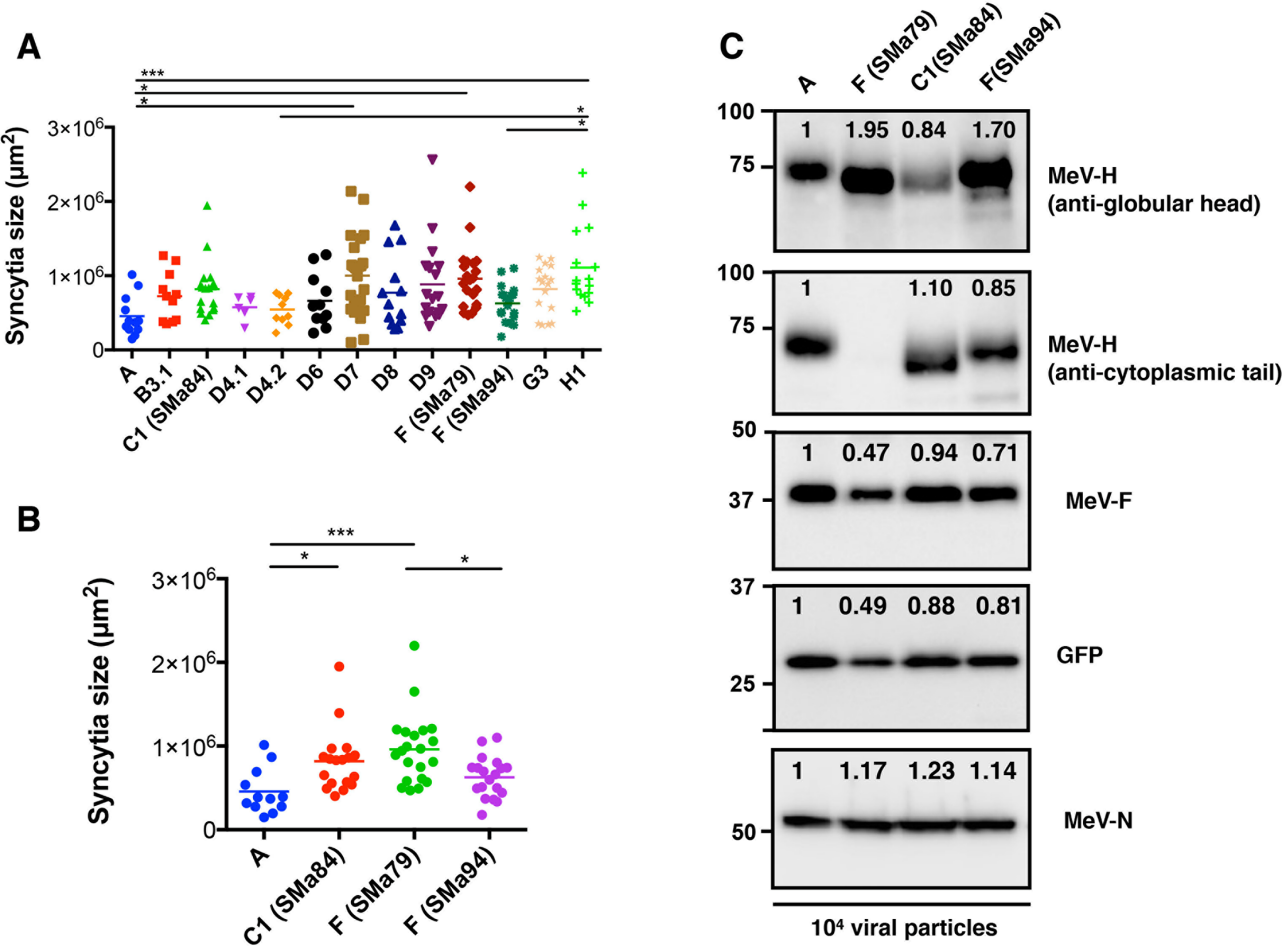
Syncytium formation of virally expressed MeV-Hs. Vero/hSLAM cells were infected with recombinant MeV expressing the indicated MeV-H. Syncytia size was measured 24 hours post transfection. Statistical significance (*P < .05; ***P < .001) was calculated by one-way ANOVA with post-hoc Tukey multiple comparisons. Differences in syncytia formation were significant between MeV-H from SSPE cases and genotype A when other wild-type genotypes were excluded from the analysis (A vs B). C, Protein composition of virus stocks. Recombinant MeVvac2(GFP)N (10^4^ plaque-forming units) possessing SSPE-specific MeV-H protein were immunoblotted with antibodies against MeV-N, MeV-H (anti-cytoplasmic and anti-globular head), MeV-F, and GFP proteins. Protein intensity was determined using a ChemiDoc Imaging System (Bio-Rad), with the MeV genotype A, set to 1, used as the comparator. Note that similar levels of MeV-H are detected when anti-cytoplasmic tail–specific antibodies are used but not when antibodies against the variable MeV-H globular are used.

Having demonstrated that the MeV-H from SSPE autopsy cases remained functional and interacted to varying degrees with the cellular receptors, we next assessed syncytium-inducing capacity in the context of the virus (ie, in conjunction with the membrane-assembly system MeV-H/MeV-F/MeV matrix protein). To this end, recombinant Moraten viruses were built and rescued in which the MeV-H gene was replaced with that from the SSPE cases. We also used recombinant Moraten viruses encoding the genotype-specific MeV-H genes we have previously described [15]. The comparative fusion activity is shown in Fig 4A, in which we measured the area of the syncytia observed 48 hours after infection of Vero/hSLAM. The virally expressed MeV-H forms showed significantly different syncytium-inducing capacity. Specifically, MeV encoding genotype H1-specific MeV-H showed superior syncytium-inducing capacity in relation to clade A (*P <* .005), genotype D4.1 (*P* < .05), and genotype F (SSPE case SMa94; *P* < .05). Even though MeV encoding genotype A-specific MeV-H can induce syncytia in Vero/hSLAM via CD46 and SLAM, viruses encoding MeV-H from SSPE SMa79 and SMa84, with SLAM as the only receptor in the cells we used, showed better syncytium-inducing capacity (*P* < .005 and *P* < .05, respectively). However, these differences were only significant when the other genotypes were excluded from the analysis (Fig 4B).

To address whether differences in fusion activity were due to differences in the incorporation of MeV-H and MeV-F proteins into virions, we assessed the protein composition of recombinant viruses by Western blot. Fig 4C illustrates that plaque-forming units (PFU) equate to similar levels of MeV nucleocapsid (MeV-N) expression. A slight decrease in the band corresponding to the green fluorescent protein (GFP) transgene, which is located upstream of the MeV-N cistron, was observed for the SSPE SMa79 virus. A comparable decrease in MeV-F expression was also detected for this virus, which argues against significant differences in expression across recombinant viruses. In the case of MeV-H expression, whereas comparable amounts were observed for MeV A, SMa84, and SMa94, the signal was absent for MeV SSPE SMa79 when we used anti-MeV-H cytoplasmic tail antibodies, as we observed before (Fig 1A). In contrast, MeV-H expression was apparently increased in the latter virus, together with the SSPE SMa94 virus, when the anti-MeV globular head mAb BH195 was used instead.

Overall, MeV-Hs from the SSPE cases were at least as fusogenic as those from non-SSPE cases and were incorporated efficiently into virions. The results of these transient-expression assays and protein binding experiments indicate that the functional activity of the viral glycoproteins is retained in the context of the virus.

### Neutralization of SSPE viruses by anti-MeV-H monoclonal antibodies

**Fig 5:**
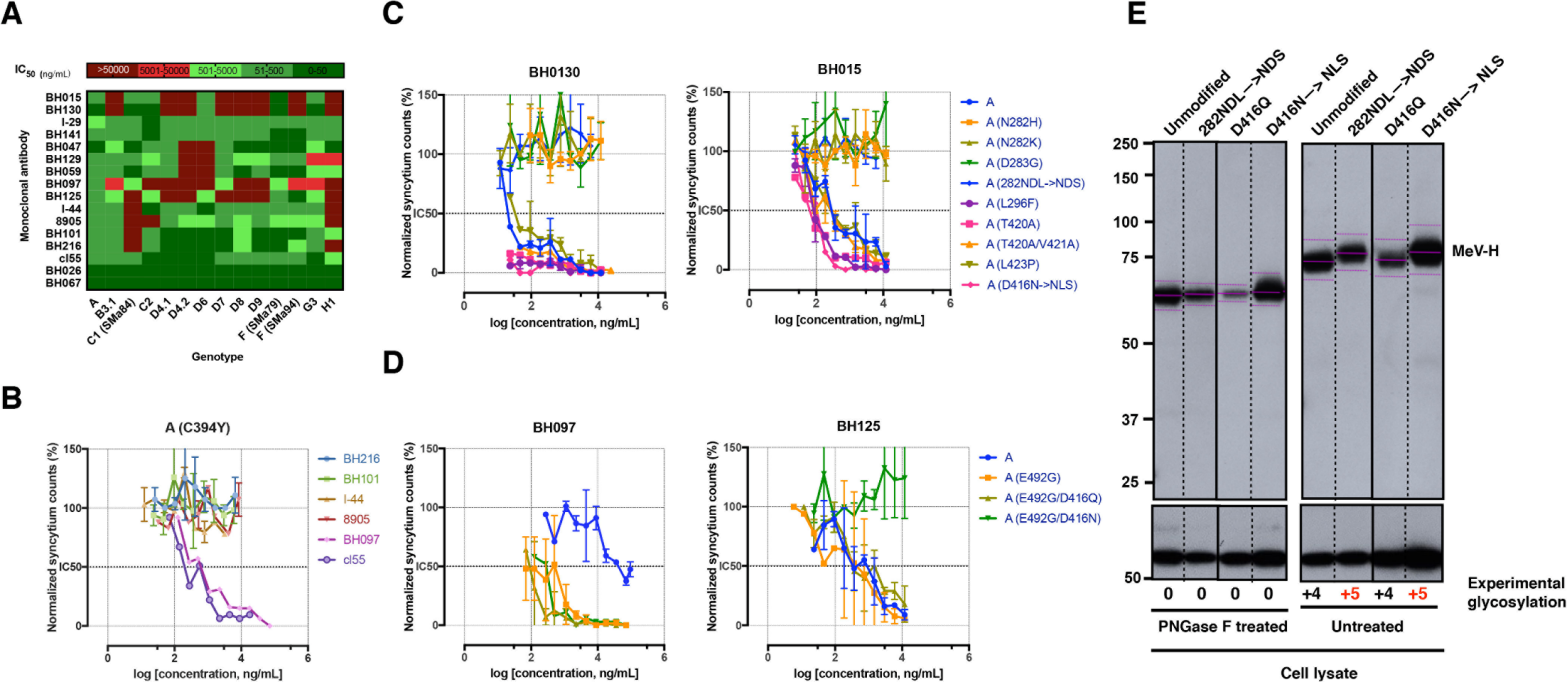
Neutralization of MeV by anti-MeV mAbs. A, Heat map representation summarizing IC_50_ values obtained from neutralization curves (see Methods) of anti-MeV-H mAbs against recombinant MeV encoding different MeV-H proteins. B, Neutralization curves for the MeV encoding MeV-H from genotype A with a C394Y point mutation. The C394Y point mutation abrogates the neutralizing activity of noose-targeting mAbs (BH216, BH101, I-44, and 8905). Antibodies BH097 (antigenic site IIa) and cl55 (antigenic site III) were used as controls for neutralization (S1 Fig). Neutralization data are presented as percentage of infection in relation to that in the absence of mAb. For each mAb concentration, points represent the mean of at least 2 independent experiments performed in quadruplicate. Error bars indicate SD. C and D, Neutralizing activity of mAbs BH130, BH030, BH097, and BH125 against different MeV mutants (indicated in parentheses). 282NDL→NLS and D416N→NLS are PNGS mutants. E, Glycosylation of PNGS mutants was confirmed by Western blot analysis of untreated and PNGase F–treated viral MeV-H proteins. For clarity in the band’s shift, the center of each band is indicated, as well as the number of PNGSs present. MeV-N was used as a loading control.

**Fig 6:**
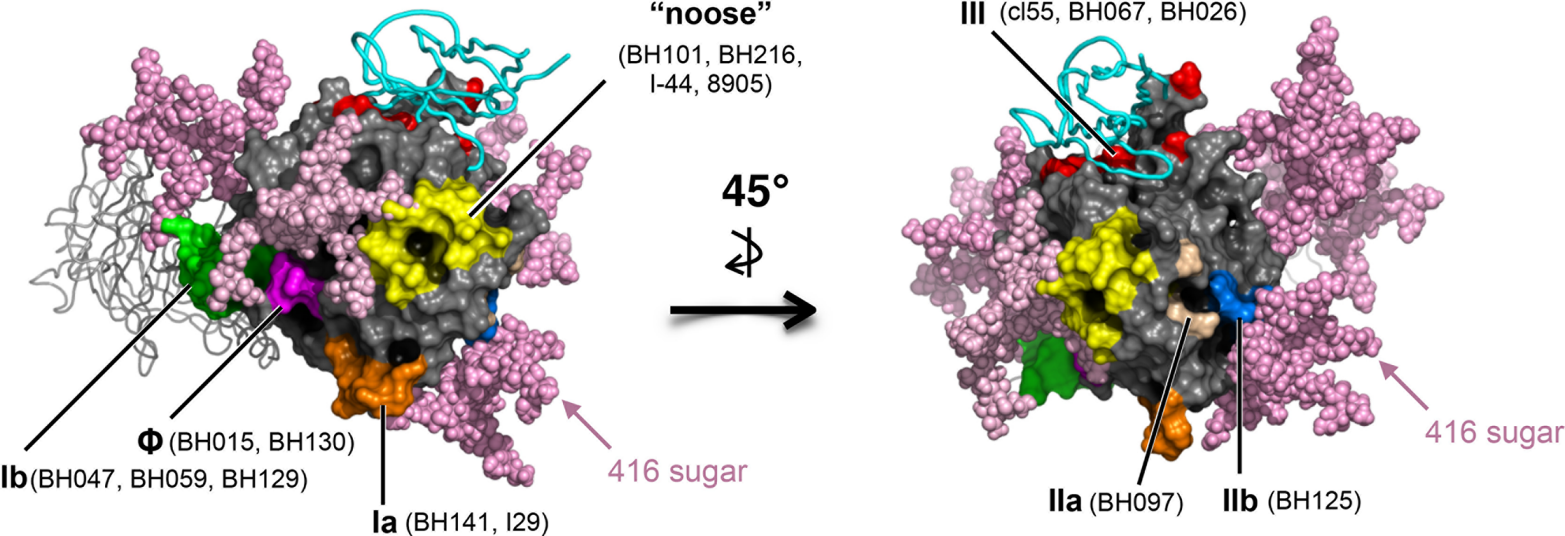
Radiographic crystallographic structure of the MeV-H globular head with modeled N-linked glycans. Surface representation of the MeV-H-SLAM complex (PBD; 3ALZ) with the SLAM receptor depicted as a cyan ribbon. For clarity, 1 monomer of the MeV-H dimer is shown in ribbon representation. Glycans (light pink spheres) attached to N168, N187, N200, N215, and N416 were modeled on the MeV-H structure using GlyProt (www.glycosciences.de). Antigenic sites displayed on the surface are color-coded. Note that the N416-linked sugar shield, which is present in most of the clade D strains, blocks binding of mAb to antigenic site IIb.

MeV can be selected in vitro to escape neutralization by multiple neutralizing antibodies [26,27]. Because patients with SSPE have increased serum levels of anti-MeV antibodies, we evaluated the SSPE MeV-H antigenic profile, together with that of other wild-type genotypes (Fig 5A). To this aim, we used a panel of anti-MeV-H neutralizing antibodies covering most of the immunogenic epitopes (Fig 6). Individual neutralization curves for monoclonal antibodies (mAbs) with recombinant MeV containing MeV-H genes of genotype A (Moraten vaccine strain), H1 (wild-type), C1 (SSPE case SMa84), and F (SSPE cases SMa79 and SMa94) are shown in the S1 Fig. mAbs targeting antigenic sites Ia (I-29 and BH141), and III (cl55, BH067, and BH026) neutralized all recombinant MeVs tested; neutralization potency was greatest at site III, followed by site Ib/NE (BH047, BH059, and BH129) and site Ia. All antigenic sites had characteristic dose-response curve slopes, except for mAb cl55 with genotype H1, in which residual resistance was observed. Neutralization heterogeneity was shown for genotype H1 when mAbs specific to the noose antigenic site were used. Whereas mAb BH101 neutralized genotype H1, mAbs BH216, 8905, and I-44 did not. None of these antibodies showed neutralization activity against the genotype C1 (SSPE SMa84) MeV, which demonstrates a complete absence of this antigenic site. However, on Western blot, a non-neutralizing noose-specific antibody, BH195, could still recognize the C1 MeV-H with efficiency similar to MeV-Hs sensitive to neutralization by other noose-targeting antibodies (Figs 1 and 4).

The noose antigenic site has a looplike structure formed by a disulfide bridge between cysteines 386 and 394 [28]. The C394Y substitution in MeV-H SMa84 would then completely destroy the structure of the antigenic site and thus facilitate a complete escape from neutralization by this class of antibodies. To confirm this, we introduced the C394Y point mutation into the MeV-H genotype A and rescued the corresponding virus. Neutralization curves showed complete resistance to noose-targeting neutralizing antibodies (BH216, BH101, I-44, and 8905), but the sensitivity to antibodies targeting different antigenic sites remained unchanged (ie, cl55 and BH097) (Fig 5B).

C1 (SSPE SMa84) virus also was not neutralized by mAbs targeting antigenic sites IIa (BH125) and IIb (BH097). This antigenic pattern was also observed for the H1 virus, which also escaped neutralization by mAbs BH015 and BH130. These 2 mAbs, together with BH097, markedly distinguish the 2 SSPE cases of genotype F (SSPE SMa79 and SMa94). These 2 viruses differed from each other in the following mutations in the MeV-H globular head: 210, 233, 283, 302, 382, 396, 423, 492, 547, 574, 575, 614, 615, and 618 (Table 2).

mAbs BH015 and BH130 also failed to neutralize genotypes B3.1, D4.1, D4.2, D7, D8, D9, and H1 (Fig 5A). To discern the antigenic determinant targeted by mAbs BH015 and BH130, we generated a panel of recombinant MeVs featuring point mutations found in resistant genotypes. We also generated an MeV possessing an additional glycosylation site at N282 (282NDL→NDS) because it was shown to escape neutralization by mAb BH015 [26]. The neutralization data indicate that BH015 and BH130 target the same, yet new, antigenic site. We designated this antigenic site “Φ” for its intermediate location between the previously defined antigenic sites Ia and Ib (Figs 5C and 6). In addition to 282NDL→NDS, the single-point mutations N282H, N282K, and D283G, which are present in genotypes D9, H1, and B3.1/F (SSPE SMa94), respectively, rendered the recombinant MeV resistant to neutralization.

We recently reported that an E492K mutation confers enhanced susceptibility to neutralization by an antigenic site IIa-targeting mAb (16CD11) [29]. Interestingly, the F (SMa79) virus possesses an E492G mutation and shows increased neutralization sensitivity to mAb BH097. To confirm that a Gly492 is responsible for this phenotype, we introduced the E492G mutation into the vaccine genotype A MeV-H gene and assessed its neutralization sensitivity. Whereas no differences in neutralization sensitivity were observed with the nearby-located antigenic site IIb-targeting antibody BH125, enhanced neutralization activity was observed with BH097 (Fig 5D). This result broadens our previous finding in which some genotypes might show enhanced sensitivity to neutralization by antigenic site IIa-targeting mAbs via point mutations at amino acid 492. Recombinant viruses with the N416-sugar shield (D416N→NLS), but not those unshielded (N416Q), escaped neutralization only by BH125 (Fig 5D and 5E). Overall, a correlation between the disease time course for SSPE (Table 1) and antigenic mutability cannot be drawn from these neutralization assay results.

### Neutralization of SSPE viruses by anti-MeV-H polyclonal sera

**Fig 7:**
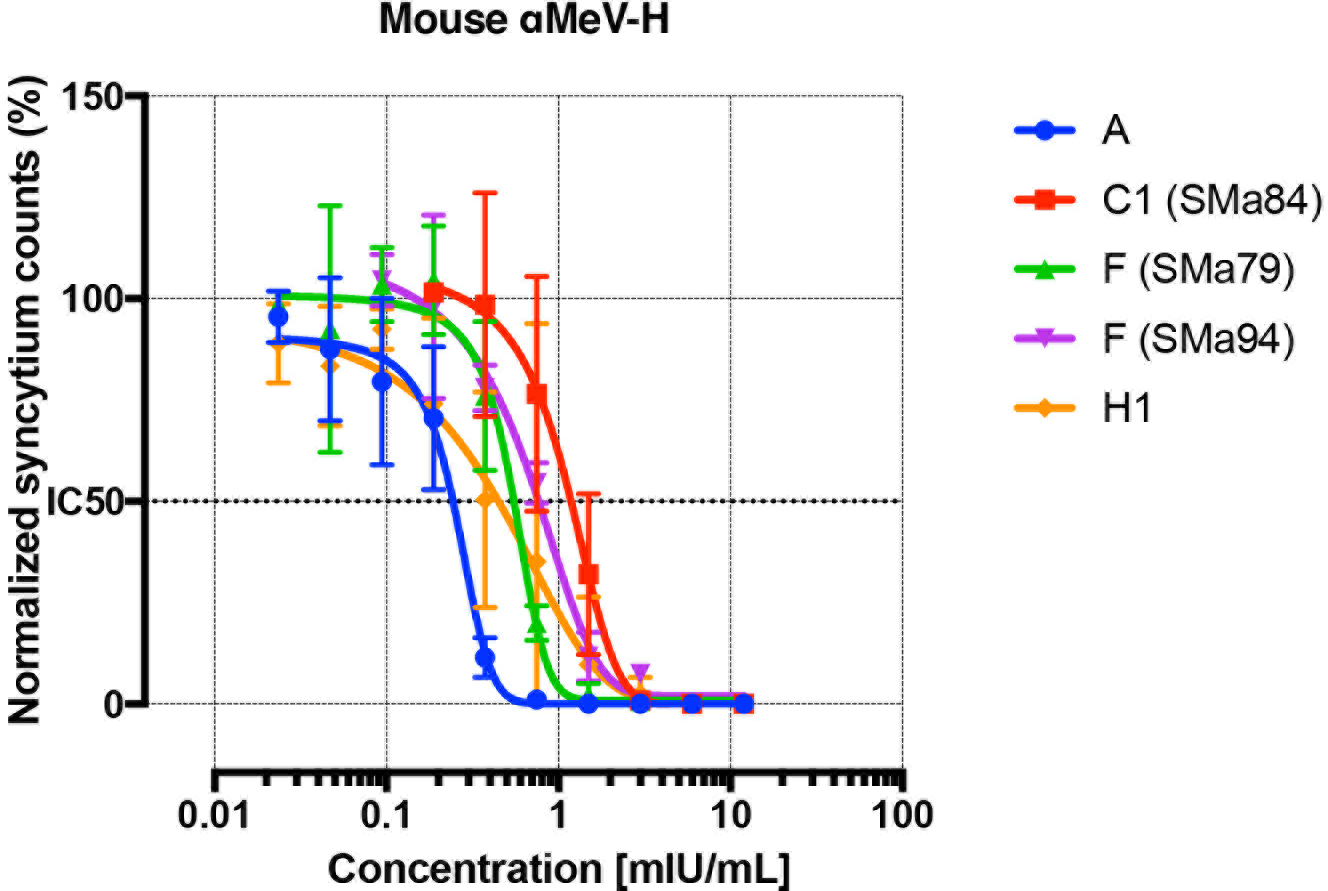
Neutralization of SSPE viruses by polyclonal antibodies. Neutralization assays were performed and data are presented as in Fig 5. Mouse anti-MeV-H polyclonal antibodies present in MeV-H genotype A–immunized mice were used for the assay. The antibody titer is standardized to mIU/mL according to the 3rd International Standard for Anti-Measles.

Virus neutralization by polyclonal antibodies could be influenced by the different neutralization efficiency of various antigenic sites, together with the presence of immunodominant antigenic sites. The mAbs previously used were derived from mice; thus, we used polyclonal anti-MeV-H mouse sera induced by vaccination with an adenovirus encoding the genotype A Edmonston strain of MeV-H. To directly measure the contribution of the antibody repertoires to virus neutralization, we used heat-inactivated mouse serum (56°C for 30 min) to assess the neutralization activity against recombinant MeVs. The results showed that no recombinant MeV escaped a polyclonal anti-MeV-H response (Fig 7). However, the neutralization curves for the SSPE forms were shifted nonsignificantly to the right in relation to A viruses (ie, decreased neutralization potency). Since H1 virus was superior in escaping multiple mAbs, the number of epitopes lacking in MeV-H does not preclude immune evasion. However, a particular pattern of sensitivity to mAb neutralization might affect the overall neutralization by polyclonal antibodies.

## Discussion

The main conclusion of the current study is that SSPE viruses are equally sensitive to neutralization as currently circulating wild-type MeV strains. Our work provides direct and concrete evidence that the MeV-H found in the brain of decedents with SSPE behaves similarly to other wild-type MeV-H forms. Antigenic change, then, does not occur over the disease course and is not a risk factor for SSPE. The conclusions are based on the following experimental evidence. First, we demonstrated that the MeV-H obtained by direct sequencing of brain autopsy material is functional despite extensive amino acid substitutions and produces cell-to-cell fusion in a receptor-binding fashion. Next, we showed that SSPE MeV-Hs efficiently interact with known wild-type MeV receptors, SLAM and nectin-4, but not with the CD46 receptor used by the live attenuated measles vaccine. Finally, we built and rescued recombinant MeVs in which the gene for MeV-H was substituted for that from SSPE cases and found that their neutralization sensitivity was similar to that of viruses coding for MeV-H from major circulating MeV genotypes. Collectively, this experimental evidence strongly supports the overall concept that the antigenic differences found in SSPE viruses are of similar magnitude to those of currently circulating MeV genotypes.

Previous studies have suggested antigenic drift as a mechanism for MeV persistence on the basis of increasing concentrations of IgG in SSPE samples collected over the disease course and differential reactivity patterns of neutralizing antibodies with SSPE strains [30–33]. These reports invariably were published many years before the identification of antigenic differences among circulating MeV genotypes [15,24,29,34–37] and were unable to address specific differences in antigenicity. At the time, SLAM/CD150 was not yet identified as the relevant MeV receptor [38] (ie, 7 years after the initial discovery of the membrane cofactor protein/CD46 as a receptor). Consequently, cells expressing CD46 were broadly used for virus isolation before it was proved to induce attenuation [39–41]. Similarly, testing whether SSPE strains develop from classical MeV strains under immune selective pressure was complicated by difficulty isolating the virus from the central nervous system [12,30]. Indeed, some SSPE viruses have been shown to be laboratory contaminants [12,42,43].

Thus, to our knowledge, this is the first MeV generated by reverse genetics with the MeV-H gene from brain autopsy samples. Different recombinant and pseudotyped viruses expressing SSPE glycoproteins have been produced [44–46], but the “SSPE strains” were passaged in Vero cells (CD46^+^, SLAM^−^, nectin-4^−^), which may have altered viral tropism. Also, the interaction with the epithelial receptor nectin-4 could not be investigated in any case [47,48]. Because our recombinant viruses were rescued in Vero/hSLAM cells (CD46^+^, SLAM^+^, nectin-4^−^), we first investigated receptor interactions by using a cotransfection expression plasmid system and direct protein-to-protein interaction with purified receptors. These experiments allowed us to address whether our reverse genetic system based on Vero/hSLAM cells introduced modifications into the MeV-H glycoproteins which may have altered the tropism.

Nectin-4 usage is crucial for the understanding of MeV pathogenesis [4,49], particularly because the neurotropism of the closely MeV–related canine distemper virus depends on nectin-4 expression patterns in the central nervous system [50,51]. Our results in tissue culture revealed nectin-4–mediated fusion of SSPE MeV-Hs, despite its weak binding with nectin-4. Another MeV-H (genotype B3.1) was also shown to weakly interact with nectin-4. We did not study the cell-to-cell fusion capacity of MeV-H genotype B3.1 in nectin-4–expressing cells, but a positive correlation between syncytium formation and attenuation was observed previously [52]. In turn, syncytia formation positively correlates with receptor binding affinity [53,54]. Of interest, MeV genotype B3.1 has been reported to be more pathogenic than genotype C2 [55]. Unfortunately, MeV-H genotype C2 was not included in our study. The results of our MeV-H-receptor binding studies should be interpreted with caution because previous studies gave discordant results [25]. Similar discrepancies have also been observed between plasmid-mediated fusion and that of recombinant viruses encoding the corresponding genes [52,56], although in these cases, interaction of the MeV matrix protein with the MeV glycoproteins seems a reasonable explanation [27].

Our failure to isolate sufficient amounts of MeV-H from genotype C1 may be due to its quaternary structure. Because the oligomeric state in the soluble form of MeV-H remains the same as in the membrane-anchored form [57], it is tempting to speculate that the lack of higher oligomeric species for MeV-H genotype C1 could have failed to compensate for low-affinity binding to the tag we used through avidity. The lack of a clear relationship between oligomeric status, surface expression, and syncytium-inducing capacity for MeV-H discouraged us from further characterization of this point [58].

Although no specific MeV genotype has been associated with a higher risk of SSPE, some MeV variants differ in the ability to establish a persistent viral infection in vitro [59,60]. To our knowledge, SSPE cases have always been caused by wild-type genotypes, and vaccine strains (genotype A) have never been encountered [61,62]. After an extensive search of the literature, we could find only genotypes C1, F, and E to be associated with SSPE cases. As shown here, wild-type MeV genotypes may be even more resistant to neutralization by mAbs than SSPE viruses. Unexpectedly, MeV genotype H1 was neutralized efficiently by the noose antigenic site–targeting mAb BH101, despite possessing several amino acid substitutions in this region [29,36].

A structural change in MeV-H in the context of the trimeric protein complex MeV-H, MeV-F, and MeV matrix protein does not convincingly explain our results since the neutralization sensitivity of our recombinant Moraten virus expressing MeV-H genotype H1 was similar to that of the primary wild-type MeV genotype H1 isolate [29]. However, changes in residues P397 and N405 instead may alter specific epitopes within the noose antigenic site rather than the whole MeV-H structure. This observation emphasizes the need to test different antibodies against the same antigenic site.

It is unclear why certain MeV genotypes adopt mutations to escape neutralization by mAbs targeting certain antigenic sites (noose, Ia, Ib, and IIb) while they also introduce mutations to enhance neutralization by otherwise poorly neutralizing mAbs (antigenic site IIa). Ndifon et al [63] observed in influenza viruses that specific amino acid substitutions in the hemagglutinin protein increased neutralization sensitivity of the escape variant by previously generated mAbs; similar phenomena have been reported in severe acute respiratory syndrome coronavirus, human immunodeficiency virus, and hepatitis C virus [64–67]. Specifically, if there is steric competition for antigen binding between antibodies with different neutralization potency, a mutation that increases binding of antibodies with low neutralizing potency would decrease the overall viral neutralization. Indeed, the MeV-H antigenic site IIa (low neutralization efficiency) is located adjacent to the noose antigenic site (high neutralization efficiency), thereby potentially leading to a decrease in viral neutralization by polyclonal mouse antibodies. This would explain the slight decrease in sensitivity of SMa79 despite being neutralized by all mAbs tested. The disruption in the structure of the noose antigenic site in SMa84 apparently had the same effect in polyclonal-mediated neutralization. The immunodominance of the noose antigenic site would then offer a plausible explanation to the shift observed in the neutralization curve with polyclonal sera.

In conclusion, although SSPE viruses show antigenic differences in neutralization by mAbs, the magnitude of such antigenic variation is similar to that found in other wild-type MeV genotypes. Therefore, it cannot be concluded that mutations of MeV-H in SSPE are a consequence of or a prerequisite for the high neutralizing antibody titers typical of SSPE cases.

## Materials and methods

An ethics board or IRB approval from Mayo Clinic is not needed since we have not conducted direct research involving human participants. We have used cloned plasmid derived from deceased patients obtained at autopsy. None of these deceased patients were from a vulnerable population and all donors or next of kin provided written informed consent that was freely given. The sequences encoded in the aforementioned plasmids have already been published and they are referenced throughout the manuscript.

### Cells

African green monkey kidney (Vero) cells and Vero/hSLAM cells [68] were cultured in Dulbecco’s modified minimal essential medium (DMEM) (HyClone; GE Healthcare Life Science) supplemented with 5% (v/v) heat-inactivated fetal bovine serum (FBS) (Gibco) and 0.5 mg/mL of Geneticin (G418; Corning). CHO cells and CHO-CD46 cells [69], a CHO cell line stably expressing human CD46, were grown in Roswell Park Memorial Institute medium (RPMI 1640; Corning) supplemented with 10% FBS. CHO cells stably expressing human SLAM [38] and CHO cells stably expressing human nectin-4 [70] were grown in RPMI 1640, 10% FBS, and 0.5 mg/mL G418. All cells were additionally supplemented with antibiotics (penicillin [100 μg/mL] and streptomycin [100 U/mL]; Gibco). All cell lines were routinely tested for mycoplasma contamination.

### Plasmids and viruses

Genes for MeV-H from SSPE autopsy cases were amplified from pGEM plasmids [18] with the pair of primers V181 (TTAATTAAAACTTAGGGTGCAAGATCATCGATAATG)/H1931 (CACTAGTGGGTATGCCTGATGT), which introduce PacI and SpeI restriction sites (underlined) flanking the MeV-H gene. The polymerase chain reaction (PCR) products were then cloned into the pCG vector and the sequences were confirmed. Mutations were introduced using QuikChange Lightning site-directed mutagenesis kit according to the manufacturer’s instructions (Agilent Technologies).

MeVvac2(GFP)N plasmid [71] was used as the backbone for the generation of the recombinant MeVs. The MeV-H gene was easily exchanged via unique PacI and SpeI sites in pCG-MeV-H plasmids. The recombinant viruses were rescued using standard protocols with modifications [72,73]. The integrity of the insert was verified through sequencing, and the corresponding virus was named according to the MeV-H gene. Genotyping was done on the sequence of MeV-H and MeV-N. The wild-type viruses used for the genotype-specific MeV-H have been previously described [15]. Virus stocks were produced upon infection of Vero/hSLAM cells at a multiplicity of infection of 0.03. Two to 3 days after infection, cells were harvested and viral particles were released by 3 freeze-thaw cycles. Viral titer was determined by plaque assay on Vero/hSLAM cells.

### Transfections, fusion assay, and protein lysates

Cells (5×10^5^) were seeded onto 6-well plates (Costar Corp) the day before the experiment. Two hours before transfection, the culture medium was removed and replaced with fresh medium without antibiotics. Then, equal amounts (1 μg) of pCG plasmids encoding MeV-F and the indicated MeV-H mutants or pCG-MeV-H alone (2 μg) were diluted in 100 μL of OptiMEM (ThermoFisher) onto a 96-well plate. Another 100 μL of OptiMEM containing 6 μL of Fugene HD (Promega) was then added to each well, and the mixture was incubated for 20 min. The solution was then added to the cells.

Fusion activity was evaluated 24 h later after Hema-Quik staining (Fisher Scientific). The size of the syncytia was quantitated using NIS-Elements microscope imaging software (Nikon). Alternatively, the number of nuclei in randomly chosen syncytia was counted.

To harvest protein lysates, cells were washed with phosphate-buffered saline (PBS) before they were lysed on ice for 15 min with 0.5 mL RIPA buffer (Abcam) containing Halt protease inhibitor cocktail (ThermoFisher). The supernatant containing the cleared lysate was collected after centrifugation at 13,000 rpm at 4°C for 15 min; aliquots were kept at −20°C if not used immediately.

### Fluorescence-activated cell sorter analysis

Expi293 cells (2.5×10^6^) (ThermoFisher) growing on 6-well plates were transfected using ExpiFectamine 293 (Gibco). At 72 h post transfection, cells were washed with cold Opti-MEM and resuspended in the same medium. Cells were then incubated on ice for 1 h with 1 μg mouse anti-MeV-H BH026 antibody. After 3 washes with Opti-MEM, cells were incubated for an additional hour with goat anti-mouse IgG (H+L) cross-adsorbed secondary antibody, Alexa Fluor 594 (A-11005; ThermoFisher). Then, cells were washed again and fixed with paraformaldehyde 1% (Electron Microscopy Sciences), and fluorescence signals were measured for 500,000 events using a FACSCanto system (BD Biosciences).

### Western blotting and N-linked deglycosylation

Samples (6.5 μL) were fractionated on NuPAGE 4%-12% Bis-Tris gels (Invitrogen). Alternatively, 10 μL of sample was incubated with or without PNGase F for 1 h at 37°C, per the manufacturer’s protocol (New England Biolabs) before fractioning. After staining with SimplyBlue SafeStain (ThermoFisher), the proteins were transferred onto a PVDF membrane using the Trans-Blot Turbo Blotting System (Bio-Rad). The membranes were blocked with 5% nonfat dry milk/tris-buffered saline (TBS) for 1 h at room temperature and incubated overnight at 4°C with the corresponding primary antibodies: anti-MeV-H cytoplasmic rabbit antipeptide serum (1:5000; made by K.W. Peng, Mayo Clinic), anti-MeV-N antipeptide serum (1:5000; [74]), mAb BH195 (1:5000) [75], anti-GFP (1:2000) (#2956; Cell Signaling Technology), or anti-β-actin-peroxidase antibody (1:25000) (A3854; Sigma). The membrane was washed 3 times with TBS containing 0.05% Tween 20 before incubation for 1 h at room temperature with the following secondary antibodies: anti-mouse (1:3000) (62–6520; ThermoFisher), or anti-rabbit (1:100000) (31462; ThermoFisher). The membranes were revealed with SuperSignal West Pico Chemiluminescent Substrate (ThermoFisher). When necessary, blot stripping was performed with Restore Western Blot Stripping Buffer (ThermoFisher) following the manufacturer’s instructions.

### Recombinant proteins

Full-length soluble ectodomains of SLAM (residues 21–237) and nectin-4 (residues 32–349) were amplified from plasmids encoding SLAM (Sino Biologicals Inc) and nectin-4 [76], respectively. The protein-specific leader sequence was substituted for the murine Ig κ-chain leader sequence, and the whole ectodomains were subcloned upstream from a cassette coding for the human engineered IgG1 Fc region, which is part of the expression vector pFUSE (pfc1-hg1e3; Invivogen). A 3C protease cleavage sequence was introduced at the 5′-end of the Fc region. MeV receptor-Fc fusion proteins were prepared from transient transfection with the Expi293 expression system (Gibco). Proteins secreted to culture supernatants were purified by affinity chromatography with a HiTrap protein G column (GE Healthcare). Stepwise elution was performed with 100 mM glycine, pH 2.7, and immediately neutralized with 1M Na_2_PO_4_, pH 9.0 (1:10). The protein size and purity was evaluated by reducing SDS-PAGE analysis and SimplyBlue SafeStain. Protein concentration was determined by a BCA protein assay (ThermoFisher).

Recombinant soluble MeV-Hs were prepared as previously described [15].

### Enzyme-linked immunosorbent assay

Precoated microtiter 96-well plates (Strep-Tactin XT, IBA GMBH) were incubated with 100 μL of supernatant containing MeV-H for 1 h at 37°C. The plates were washed 4 times with PBS containing 0.05% Tween 20 (PBS-T). MeV receptor-Fcs or anti-FLAG M2 antibody (Sigma-Aldrich) were serially diluted in buffer (Tris/HCl 10 mM, pH 7.5; NaCl 150 mM) and then transferred to the MeV-H–coated plates for 1 h at 37°C. The plates were washed again 4 times with PBS-T and incubated for 1 h at room temperature with anti-human IgG (Fc-specific) horseradish peroxidase (HRP) antibody (1:70000) (A0170; Sigma-Aldrich) or anti-mouse IgG (H+L)-HRP (1:2000) (62–6520; ThermoFisher) antibody. After developing for 15 min with 1-Step Ultra TMB (ThermoFisher), the reaction was stopped with 2M H_2_SO_4_. The plates were read at an optical density of 490 nm using an Infinite M200 PRO multimode microplate reader (Tecan).

### Antibodies and virus neutralization assay

Mouse anti-MeV-H antisera was generated by immunization with adenovirus expressing the genotype A Edmonston strain MeV-H, as described previously [26].

Monoclonal MeV-H–specific antibodies used in this study have been characterized previously by ELISA binding based on biotinylated overlapping peptides: BH101, BH216, BH047, BH059, BH129 [75,77], BH026, BH067, BH125, BH015 [78]; and/or escape mutant viruses: BH141 [26], BH097 [79], BH047, BH059 and BH129 [15]; I29 [80], cl55 [81], I44 [26,80] and mAb8905 [29].

Neutralization assays were performed in quadruplicate, and the experiment was repeated at least 2 times depending on the antibody. For that, Vero/hSLAM cells were seeded onto 96-well plates at a density of 10^4^ cells/well in 100 μL and were incubated at 37°C overnight. Anti-MeV-H antibodies were serially diluted 2-fold in Opti MEM and incubated with an equal volume of virus diluted to 90–120 PFU per 150 μL. The virus-antibody mixture was incubated for 1 h at 37°C before being transferred to the cells. After 90 min of infection, DMEM-5% FBS media was added to each well. Plates were incubated for 48 h, and the number of enhanced green fluorescent protein–expressing syncytia/well was counted using a fluorescence microscope. This experiment was repeated at least 2 times depending on the mAb.

### Statistical analysis

GraphPad Prism 6 for Mac (www.graphpad.com) was used for all statistical analysis. One-way ANOVA with post-hoc Tukey multiple comparisons was used to compare multiple groups. IC_50_ was calculated by nonlinear regression of the virus-neutralization curves.

## Supporting information

S1 Fig Neutralization activity of various mAbs against SSPE viruses. Recombinant MeVs coding for different MeV-Hs were incubated with the indicated mAb for 1 h at 37°C before infection of Vero/hSLAM cells. The number of infected green fluorescent protein–expressing foci per well were counted 2 days post infection and expressed as a percentage of the number of infected foci in the absence of mAb.
(TIF)

## Abbreviations

CHO: Chinese hamster ovary
DMEM: Dulbecco’s modified minimal essential medium
FBS: fetal bovine serum
GFP: green fluorescent protein
HRP: horseradish peroxidase
mAb: monoclonal antibody
MeV: measles virus
MeV-H: MeV envelope hemagglutinin protein
MeV-F: MeV envelope fusion glycoprotein
MeV-N: MeV nucleocapsid
PBS: phosphate-buffered saline
PBS-T: PBS containing 0.05% Tween 20
PCR: polymerase chain reaction
PFU: plaque-forming units
PNGase F: peptide-N-glycosidase F
PNGS: potential N-linked glycosylation site
RPMI: Roswell Park Memorial Institute medium
SLAM: signaling lymphocytic activation molecule
SSPE: subacute sclerosing panencephalitis
TBS: tris-buffered saline
Vero/hSLAM: Vero cells expressing human SLAM
